# Affiliative Behavior, Ultrasonic Communication and Social Reward Are Influenced by Genetic Variation in Adolescent Mice

**DOI:** 10.1371/journal.pone.0000351

**Published:** 2007-04-04

**Authors:** Jules B. Panksepp, Kimberly A. Jochman, Joseph U. Kim, Jamie J. Koy, Ellie D. Wilson, QiLiang Chen, Clarinda R. Wilson, Garet P. Lahvis

**Affiliations:** 1 Neuroscience Training Program, University of Wisconsin, Madison, Wisconsin, United States of America; 2 Department of Surgery, University of Wisconsin, Madison, Wisconsin, United States of America; 3 Undergraduate Program in Religious Studies, University of Wisconsin, Madison, Wisconsin, United States of America; 4 Undergraduate Program in Biological Sciences, University of Wisconsin, Madison, Wisconsin, United States of America; 5 Waisman Center for Developmental Disabilities, University of Wisconsin, Madison, Wisconsin, United States of America; Centre National de la Recherche Scientifique, France

## Abstract

Social approach is crucial for establishing relationships among individuals. In rodents, social approach has been studied primarily within the context of behavioral phenomena related to sexual reproduction, such as mating, territory defense and parental care. However, many forms of social interaction occur before the onset of reproductive maturity, which suggests that some processes underlying social approach among juvenile animals are probably distinct from those in adults. We conducted a longitudinal study of social investigation (SI) in mice from two inbred strains to assess the extent to which genetic factors influence the motivation for young mice to approach one another. Early-adolescent C57BL/6J (B6) mice, tested 4–6 days after weaning, investigated former cage mates to a greater degree than BALB/cJ (BALB) mice, irrespective of the sex composition within an interacting pair. This strain difference was not due to variation in maternal care, the phenotypic characteristics of stimulus mice or sensitivity to the length of isolation prior to testing, nor was it attributable to a general difference in appetitive motivation. Ultrasonic vocalization (USV) production was positively correlated with the SI responses of mice from both strains. Interestingly, several USV characteristics segregated with the genetic background of young mice, including a higher average frequency and shorter duration for the USVs emitted by B6 mice. An assessment of conditioned place preference responses indicated that there was a strain-dependent difference in the rewarding nature of social contact. As adolescent mice aged, SI responses gradually became less sensitive to genetic background and more responsive to the particular sex of individuals within an interacting pair. We have thus identified a specific, genetic influence on the motivation of early-adolescent mice to approach one another. Consistent with classical theories of motivation, which propose a functional relationship between behavioral approach and reward, our findings indicate that reward is a proximal mechanism through which genetic factors affect social motivation during early adolescence.

## Introduction

Social interactions constitute a broad range of forms that are responsive to the particular environmental or social context where they occur. Although food availability [1]–[3] disease prevalence [4]–[6] and seasonal changes [7], [8] can influence the level of sociability among conspecifics, perhaps more familiar are the effects of group structure on social interaction [9]–[15]. For example, an adult female will perform a remarkable range of social behaviors throughout her life, ranging from courtship to territorial defense to maternal care, all of which are contingent upon her social context, individual status and age. It is axiomatic that social interactions among adults are closely tied to reproductive opportunities [16]. However, juvenile animals lack reproductive competence [17]–[19], so it is conceivable that some aspects of the juvenile social repertoire are sensitive to motivational variables distinct from those that operate during adulthood.

Social interaction has been extensively described among juvenile animals in the laboratory [20]–[24], as well as in more naturalistic contexts [8], [25]–[30]. Compared to adults, social interactions between juveniles usually have a more playful quality [31]–[33]. During pubertal and adolescent development [18], [34], [35], these patterns of social behavior transition into forms that are more typical of adults [35]–[37], yet very little is known about the interplay between motivational variables and the expression of approach behavior as it changes throughout adolescence. For example, strong parent-offspring bonding and gregariousness among kin, which are common features of sociality among juvenile mammals, can dissipate during adolescent development. As adolescent animals near adulthood, they begin to express motivations to disperse and integrate into new social groups, to establish a territory and interact with potential mates, and ultimately to reproduce and care for offspring [28], [38], [39]. This reallocation of social interests is the cumulative product of a variety of factors, including interactions between endocrine physiology [17], [40], [41], social experience [42], [43] and brain anatomy [44], [45]. Adolescence is thus a period of dynamic social transformation in which juvenile behavior is organized to face new challenges that accompany being an adult.

A recent series of studies [46]–[48] demonstrated that prepubescent mice from the B6 strain are particularly pro-social, whereas age-matched BALB mice are much less responsive to social opportunities. Specifically, these studies illustrated that adolescent B6 mice approach and investigate social stimuli to a greater extent than BALB mice [46], [47]. Interestingly, adult mice from these two genetic backgrounds appear to be much less distinct than juvenile mice in terms of their motivation to approach and investigate conspecifics [49], [50], although such a comparison has not been thoroughly tested. In the present study, we evaluated patterns of social interaction within pairs of BALB and B6 mice throughout adolescent development, in conjunction with measurements of mounting behavior, investigation of a novel olfactory stimulus, food consumption, ultrasonic communication and social conditioned place preference during early adolescence. Overall, our results demonstrate that genetic variation has a direct impact on the expression of social approach and the production of ultrasonic vocalizations by early-adolescent mice, as well as on the underlying reward value they assign to the opportunity for social contact. Importantly, the strain-dependent influence on social approach was most pronounced during early adolescence and diminished with the appearance of gender-specific social interests during later stages of adolescent development. We thus propose that specific alleles interact with a *social reward* process in the early-adolescent mouse, modulating the value of social interaction, and thereby influencing the degree to which conspecifics are motivated to approach each other.

## Materials and Methods

### Mouse husbandry

Mice from the BALB/cJ (BALB) and C57BL/6J (B6) strains were purchased from Jackson Laboratories (Bar Harbor, ME, USA) and subsequently bred at the University of Wisconsin, Biotron (Madison, WI, USA) under tightly controlled temperature (21±1°C), humidity (range, 50–60%) and light (14∶10 hr light/dark, ‘lights off’ from 1130–2130) conditions. To reduce potential influences from genetic drift, new mice were routinely introduced to the breeding colony and brother-sister matings were avoided. Mice were maintained under specific-pathogen free conditions and housed in standard polyurethane cages (290×180×130 mm) that contained 1/8” grain-size corncob bedding (The Andersons, Maumee, OH, USA) and nesting material (Ancare Corporation, Bellmore, NY, USA), with *ad libitum* access to food (Teklad Rodent Diet, Harlan, Madison, WI, USA) and water. Pregnant females were isolated and pups were weaned on postnatal day (PD) 20–21 (day of birth = PD 0). Animal care and experimental protocols were conducted in accordance with the regulations of the institutional care and use committee at the University of Wisconsin - Madison and the NIH *Guide for the Care and Use of Laboratory Animals*. Our own laboratory personnel carried out all aspects of the mouse husbandry under strict guidelines to insure gentle and consistent handling of the mice.

### Social interaction tests: general methodology and measurements

**Figure 1:**
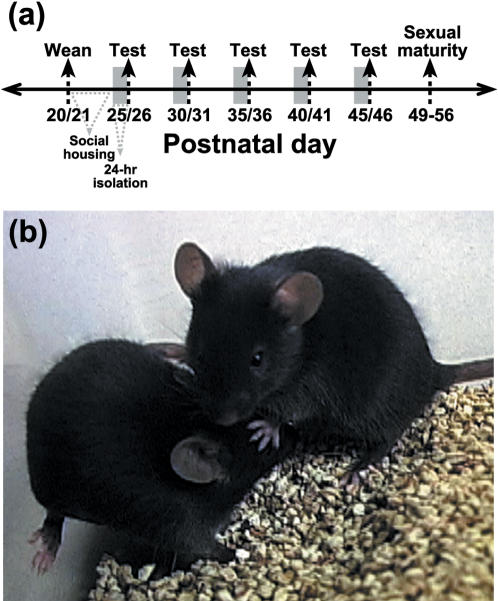
Social approach among adolescent mice. (a) The SI responses of test mice were quantified during 5-min tests throughout adolescent development. Weaning age and the average age of reproductive maturity in females is illustrated for mice from both strains. (b) Photograph of a B6 mouse investigating a former cage mate after 24 hrs of social isolation.

At weaning, mice were formed into a social group (2 mice from each sex) that served as the general housing arrangement for all subsequent tests, with the exception of *Experiment 4* (see below). Sibling number, sex bias and maternal experience (primiparous vs. multiparous) were recorded for each litter and a series of statistical tests for correlation indicated that these variables were not related to the social responsiveness of adolescent mice (data not shown), and thus were not considered further. Twenty-four hours prior to testing, each mouse from a social group was isolated within a clean cage that contained fresh corncob bedding without nesting material. After 24 hrs of social isolation, two mice from each group were randomly designated as ‘test’ mice, while the remaining individuals served as ‘stimulus’ mice. Testing entailed placing a stimulus mouse into the cage where an isolated test mouse had resided for the previous 24 hrs and then monitoring the amount of social investigation (SI) that test mice directed towards the stimulus mouse during a 5-min period (see **Figures 1a & 1b**). Behavioral variables included: [i] sniffing or snout contact with the head/neck/mouth area, [ii] sniffing or snout contact with the flank area, [iii] direct contact with the anogenital area, [iv] social pursuit within one body-length as the stimulus mouse moved continuously throughout the cage and [v] social grooming. These variables were highly correlated and combined into a composite measure of SI. We also recorded additional features of social interaction, such as social proximity (i.e., mice within one-body length of each other without movement or direct contact) and ‘jerk-and-run’, a play-like behavior (see ref. [30]), but these behaviors were infrequent and highly variable among pairs of mice, and therefore not considered in the comparisons of SI. Mounting behaviors were also observed during some of the SI tests and recorded as a categorical variable. These behavioral sequences were very brief, usually entailing physical contact that was not conducive for copulation, and were not included in the composite measure of SI. In a set of preliminary experiments, where the testing period lasted 10 min, observations of tail rattling and wrestling behavior were included in the ethogram. The social behaviors of stimulus mice were also noted, but not included in the behavioral analyses.

SI testing was conducted during the dark phase (1300–1900), under dim red illumination, in a sound-dampened room that was structurally identical to the mouse colony. Mouse cages were transported approximately 5 meters from the colony, through a dimly-lit intervening room, to the procedure room >30 min prior to testing. The top from the cage containing a test mouse was replaced with a 1/8”-thick piece of transparent Plexiglas® 5–10 min before testing began. Following this habituation period, a stimulus mouse was introduced to the side of the cage opposite to the test mouse. Behaviors were videotaped (Sony, DCR-VX2100, Japan) and stored on a Dell Pentium IV PC for additional analysis. All behaviors were scored in duplicate with the aid of computer-assisted analysis software (ButtonBox v.5.0, Behavioral Research Solutions, Madison, WI, USA), once during the experiment and once during a subsequent ‘off-line’ analysis session by a different observer that was blind to the age and gender of the interacting mice. The presentation of all SI data and statistical outcomes in this study are based on an average of these two independent measurements (inter-rater reliability, Pearson's correlation, r = 0.93, d.f. = 639).

### Social interaction tests: experimental design

#### Experiment 1–Effects of genotype and sex composition within a dyad on SI throughout adolescent development

Mice were weaned on PD 20/21 and housed in a mixed-sex social cohort containing 4 siblings. The identities of test and stimulus mice within a social group were determined randomly on the first day of SI testing and remained constant for all subsequent tests. SI testing was conducted at 5-day intervals on PD 25/26, PD 30/31, PD 35/36, PD 40/41 and PD 45/46 (**Fig. 1a**). Stimulus mice were identified by a small mark placed at the base of the tail with a permanent marker. On average, SI tests were carried out at 4 of the 5 developmental time points for each pair of mice. Following each testing period, all mice were weighed, placed into a clean cage with fresh bedding and nesting material, returned to the colony room, and left undisturbed until the next 24-hr isolation period. The SI responses of male and female test mice were measured in response to stimulus mice from both sexes. For tests that involved a male and female, sample sizes were 12–19 test mice per sex combination at each age (mice were derived from 18 litters per strain). For tests that involved same-sex pairings, sample sizes were 10–11 test mice per sex combination at each age (mice were derived from 10 litters per strain).

#### Experiment 2–Influence of maternal care on strain differences in SI for early-adolescent male mice investigating female mice

BALB (n = 4) and B6 (n = 3) litters were cross-fostered to a postpartum dam from the alternate strain within 12 hrs of birth. Subsequent care for the mice was consistent with Experiment 1. At weaning, four siblings (2 per sex) were formed into a social group and left undisturbed until social isolation on PD 29/30, as previously described. On PD 30/31, male test mice were tested for SI with a familiar stimulus female of the same strain. N's = 10 test mice per strain.

#### Experiment 3–Early-adolescent BALB and B6 male mice investigating female mice from the opposite strain

At weaning, two males from one strain were housed with 2 females from the alternate strain. Mouse husbandry and SI testing was performed as described for Experiment 2. Following 8–10 days of continuous housing in these social groups, all mice were socially isolated for 24 hrs and then tested for SI. A 5-min social interaction test was performed with male test mice (PD 30/31) approaching a familiar, age-matched female from the alternate strain. N's = 16 BALB and 14 B6 test mice from 5–6 litters per strain.

#### Experiment 4–Early-adolescent BALB and B6 male mice investigating females after long-term social isolation

At weaning, individual male mice were placed in isolate housing and left undisturbed for 8–10 days until SI testing. All mice were socially isolated into a clean cage 24 hrs before SI testing. A 5-min social interaction test was performed with male test mice (PD 30/31) approaching an unfamiliar female from the same strain. N's = 8 BALB and 9 B6 test mice from 4 litters per strain.

#### Experiment 5–Early-adolescent BALB and B6 mice investigating a novel olfactory stimulus

At weaning, mice were formed into mixed-gender social groups and maintained as previously described. Mice were socially isolated into a clean cage 24 hrs before testing. On PD 30–36, mice were presented with a cotton ball scented with lemon extract (500 µl) and olfactory investigation was measured for 5 min. Olfactory investigation included contacting, actively manipulating or sniffing (<10 mm) the cotton ball. Mice were re-grouped into a clean cage following testing. N's = 13–15 mice per sex for each strain.

#### Experiment 6–Early-adolescent BALB and B6 mice approaching and consuming a familiar food source after food deprivation

Forty-eight hours after testing for investigation of a novel olfactory stimulus, mice from Experiment 5 were re-isolated into a clean cage and food-deprived during a 24-hr social isolation period. On PD 32–38, each mouse was provided with a single pellet of standard lab chow on the floor of its cage and the total amount consumed within a 10-min period was measured. N's = 13–15 mice per sex for each strain.

### Measurement and characterization of ultrasonic vocalizations during social interaction

Ultrasonic vocalizations (USVs) were recorded for all SI tests conducted during Experiment 1. An ultrasound microphone (UltraSoundGate model CM16, Avisoft Bioacoustics, Berlin, Germany) with a 10–180 kHz flat-frequency range was lowered to the plane of the cage top, where there was a small opening (30-mm diameter) centrally located within the Plexiglas® cage cover. USVs were collected with an UltraSoundGate 116 acquisition system and the Avisoft-Recorder v.2.97 (Avisoft Bioacoustics), and stored as wav files for subsequent analysis. For practical purposes, quantitative analyses of USV emission were focused on SI tests conducted on PD 30/31, to compare with our previous work [51] that was conducted during this particular period of adolescent development.

Five sonograms, corresponding to the first 15-sec interval of each minute during a SI test, were generated for all pairs of mice tested on PD 30/31 and subjected to an extensive quantitative analysis (SASLab Pro v.4.39, Avisoft Bioacoustics). A 40-kHz band-pass filter was used to minimize background noise during recordings; however, most wav files still contained a considerable amount of ‘non-USV’ signal that compromised the accuracy of the automated parameter-measurement functions available within the SASLab Pro software format. Thus, extraneous noise was identified and removed from all of the sonograms. When a rater found an ultrasound signal that was difficult to interpret, the call was evaluated by a minimum of one additional, trained observer and identification required a consensus by all raters. Each sonogram was then evaluated with a series of automated parameter measurements that tallied the total number of USVs produced, USV duration, the average dominant frequency of a USV and the inter-vocalization interval.

Since a considerable amount of frequency modulation existed within individual USVs, a quantitative framework was devised to reliably identify USV subtypes based on their distinct sonographic characteristics (see Results). Two blind observers categorized each USV into a particular subtype, and the presentation of all data and statistical outcomes in the present study are based on average of these duplicate measurements (inter-rater reliabilities, Pearson's correlations, r = 0.90–0.98 for each USV subtype). Additionally, random samples of each USV subtype were subjected to a more detailed quantitative analysis. USVs from each subtype were sampled from a set of 15-sec sonogram files that represented ≥5 mouse pairs per sex combination for each strain. Sample sizes ranged from 32–156 syllables per USV category for each strain. In addition to measuring the duration and average pitch of these calls, the pitch of each syllable was sampled at 1-msec intervals, which permitted a higher temporal resolution for analyzing internal frequency changes within individual USVs.

### Social conditioned place preference

#### Experiment 7–Social reward in early-adolescent BALB and B6 mice

Mice were weaned on PD 20/21, maintained in social groups as previously described and left undisturbed until the conditioning phase of the experiment. On PD 25–30, the day prior to conditioning, mice were socially isolated at 1400–1600 into a clean cage that contained corncob bedding and nesting material. Social conditioning took place over the next 3 days, which included 2 conditioning sessions per day (conditioning sessions occurred at 0900–1100 and 1900–2100 each day). During a conditioning session, mice from each social group (2 same-strain mice per sex) were either reunited or socially isolated for 30 min in one of two distinct environments (aspen or paper bedding) situated within a single compartment of a 3-chambered testing arena (for additional details see ref. [51]). Unconditioned mice from the control groups were socially isolated during all 30-min conditioning sessions. To ensure that mice in the control groups received an equivalent amount of experience with the unconditioned stimulus (i.e., social interaction), they were reunited with their respective social group once per day for 30 min in a novel, plastic enclosure (380×200×155 mm) that was lined with corncob bedding and situated within a procedure room distinct from where the conditioning sessions were conducted. All variables associated with the conditioning procedure were randomized and counter-balanced across the unconditioned and conditioned groups of mice.

On the final day of conditioning, individual mice were allowed to freely explore a 3-compartment testing arena (300×150×150 mm per compartment) for a 15-min habituation period with no conditioning cues present (habituations took place at 1400–1600 under dim red light). On the test day (PD 30–35), an individual mouse was placed in the central compartment of the testing arena (where no conditioning cues were present) and its movement throughout the arena was videotaped for a 30-min period. The time spent in each compartment (peripheral compartments contained the socially-paired and isolation-paired beddings, respectively) and the number of transitions made between each compartment was quantified during a subsequent off-line analysis. Preference scores were calculated as the duration a mouse spent in the aspen bedding-lined compartment *minus* the duration spent in paper bedding-lined compartment. Ten BALB mice (4 and 6 individuals from the conditioned and unconditioned groups, respectively) were not included in the statistical comparisons due a greatly reduced level of exploratory activity, as detailed in ref. [51]. N's = 28–32 mice per strain for each of the unconditioned and conditioned groups.

### Statistical analyses

A 2×4×5 analysis of variance (ANOVA), with the strain and sex combination of mouse pairs as between-group factors and age as a repeated measure, was used to analyze the amount of SI expressed by test mice during Experiment 1. Orthogonal contrasts (JMP v.6.0, SAS Institute Inc., Cary, NC; also see ref. [52]) were used to evaluate the presence of strain differences at each sampling point during adolescent development and within-strain differences for PD 25/26 vs. PD 45/46 mice. For Experiments 2–4, one-factor ANOVAs were used to assess the effect of genotype on SI. For Experiments 5 and 6, two-factor ANOVAs (with strain and sex as between-groups factors) were used to evaluate olfactory investigation and food consumption, respectively. For Experiment 7, a 2×6 ANOVA (with sex and conditioning group as between-groups factors) was used to assess the place-preference scores. Tukey's honestly significant different (HSD) tests and orthogonal contrasts were employed to conduct post-hoc comparisons for Experiments 6 and 7, respectively. A majority of the USV data was assessed with 2×4 ANOVAs (with the strain and sex combination of mice within an interacting pair as between-group factors) and nested orthogonal contrasts when appropriate. For variables that were not normally distributed, non-parametric χ^2^-approximations were used to evaluate differences in the median value between strains. Linear regressions, Pearson's correlations and contingency-table analyses were conducted as necessary (tests involving correlation matrices were corrected for multiple comparisons). For all statistical tests, P<0.05 was considered significant.

## Results

### Strain-dependent variation in social investigation during adolescent development:

**Figure 2:**
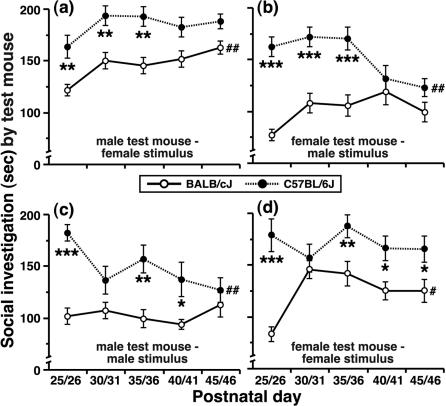
Differences in social investigation between adolescent mice as a function of genetic background, age and sex. SI responses of BALB and B6 test mice during adolescent development for (a) males approaching females, (b) females approaching males, (c) males approaching males and (d) females approaching females. All data are presented as the mean±SEM (* P<0.05, ** P<0.01, *** P<0.001 for BALB vs. B6 mice; ^#^ P<0.05, ^##^ P<0.01 for PD 25/26 vs. PD 45/46 mice).

We quantified the amount of SI that test mice direct towards familiar conspecifics placed within their home cage during a 5-min period at multiple points throughout adolescent development (see **Figure 1a** & and *Experiment 1* in Materials and methods). Following 24 hrs of isolation, social reunion with a stimulus mouse resulted in a larger SI response (**Fig. 1b**) by test mice from the B6 strain compared to age-matched BALB mice (**Figs. 2a–d**; main effect of genotype, F [1,260] = 61.1, P<0.0001). On PD 25/26, which was the first day of SI testing, this strain-dependent difference was evident for all test-by-stimulus mouse combinations; i.e., males approaching females (M-F pairs; **Fig. 2a**), females approaching males (F-M pairs; **Fig. 2b**), males approaching males (M-M pairs; **Fig. 2c**) and females approaching females (F-F pairs; **Fig. 2d**).

The social responsiveness of adolescent mice was noticeably variable across adolescent development (main effect of age, F [4,260] = 2.3, P = 0.06). Moreover, strain- and sex-dependent effects on SI also changed during adolescence (P<0.01 for both interaction effects, F-statistics not shown) and interacted with each other as a function of age (genotype×sex×age interaction, F [12,260] = 2.6, P = 0.003). This three-way statistical interaction corresponded to distinct effects of the sex of interacting mice on SI responses, which gradually came to overshadow the strain-dependent influence during late adolescence. For example, BALB test mice expressed larger SI responses towards a *female* stimulus mouse on PD 45/46 compared to mice on PD 25/26 (**Figs. 2a & 2d**), corresponding to a decline in the strain difference that occurred during early adolescence. Older test mice (PD 45/46) from the B6 strain expressed diminished SI responses, relative to early-adolescent mice (PD 25/26), when they were reunited with a *male* stimulus mouse (**Figs. 2c & 2b**). On PD 45/46, strain-dependent variation in SI was evident only for female test mice that had been paired with another female and this strain difference was considerably smaller than the difference that was found for the same pairs on PD 25/26 (see **Figure 2d**).

As adolescent test mice matured, we noted several instances of social approach that were distinct from those included in the composite measurement of SI (**Table 1**). For example, a proportion of B6 males (PD 30/31) began to direct mounting behaviors towards female stimulus mice approximately 5 days earlier than male BALB mice (PD 35/36). These behavior patterns usually encompassed 2–3 successive mounting attempts (<5 sec) that occurred during the final 60 sec of the testing period. Mounting was disproportionately expressed by male test mice in response to the introduction of a female, accounting for 81% of all occurrences, and was targeted towards both the posterior and anterior portion of the stimulus mouse. The likelihood that mounts occurred during M-F interactions increased as a function of age (χ^2^ = 32.5, d.f. = 4, P<0.0001) and there was a general trend for male test mice from the B6 strain to engage in more mounting behaviors than age-matched BALB males at all sampling points other than PD 25/26 (see **Table 1**; χ^2^ = 8.3, d.f. = 1, P<0.01).

**Table 1:** Proportion of male test mice that displayed mounting behavior towards a female stimulus mouse.

Age	BALB/cJ	C57BL/6J
PD 25/26	n = 16 (n/a)	n = 18 (n/a)
PD 30/31	n = 16 (n/a)	5 of 19 (26%)
PD 35/36	3 of 12 (25%)	9 of 16 (56%)
PD 40/41	6 of 17 (35%)	9 of 13 (69%)
PD 45/46	5 of 18 (28%)	9 of 18 (50%)

We did not observe any instances of aggressive behavior between adolescent mice during the 5-min SI tests. However, during a preliminary set of experiments, which entailed 10-min testing sessions, approximately 35% of BALB test males (PD 39–42) engaged in wrestling or tail rattling when they were paired with another male, whereas age-matched M-M pairs from the B6 strain never expressed such patterns of behavior (J.B.P & G.P.L., personal observations). Importantly, all forms of ‘adult-like’ behavior were observed before females had exhibited patent vaginal swelling, lordosis or estrus (see www.jax.org/phenome). Furthermore, the development of mounting and aggressive behaviors by male test mice occurred on a similar timeframe as the appearance of gender-effects on SI. When male test mice that had exhibited mounting attempts were excluded from the statistical analysis (data not shown), differences between the average SI responses of mice for each strain-by-age comparison were not distinguishable from those presented in **Figure 2a**.

**Figure 3:**
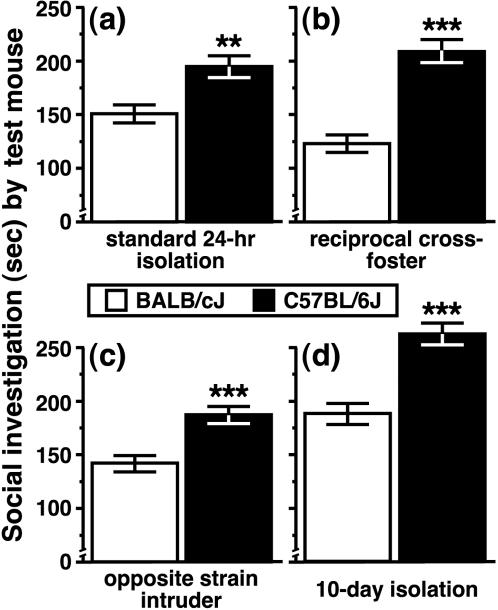
Strain-dependent differences in social investigation as a function of maternal care, stimulus mouse characteristics and length of social isolation. (a) Following 24 hours of social isolation, on PD 30/31, male B6 mice investigated a familiar female stimulus mouse for a greater duration then age-matched BALB males. This strain-dependent pattern was also expressed by (b) male mice that had been raised by a mother of the alternate strain, (c) male mice approaching a female from the alternate strain and (d) male mice approaching a same-strain female after 8–10 days of social isolation. All data are presented as the mean±SEM (** P<0.01, *** P<0.001).

To determine whether the strain difference in SI between early-adolescent mice was attributable to the genetic background of the test mouse, we conducted a series of control experiments in which PD 30/31 males were given an opportunity to investigate age-matched females after 24 hrs of social isolation. The strain-dependent difference in SI (data for the PD 30/31 mice plotted in **Figure 2a** are presented again in **Figure 3a**) was not altered when BALB and B6 mice pups had been raised by a mother from the opposite strain and then housed with siblings from weaning until testing (see *Experiment 2*; **Fig. 3b**; F [1,18] = 39.3, P<0.0001). Furthermore, the strain difference was also present for mice that were maintained in mixed-strain social housing for 8–10 days prior to testing (see *Experiment 3*; **Fig. 3c**; F [1,28] = 21.3, P<0.0001), suggesting that differences between the specific contexts associated with BALB and B6 housing could not account for strain-dependent variability in SI. In another experiment, we found that a longer period of social isolation (8–10 days) did not affect the strain-dependent pattern in SI between early-adolescent mice from the BALB and B6 strains (see *Experiment 4*; **Fig. 3d**; F [1,14] = 28.7, P<0.0001).

**Figure 4:**
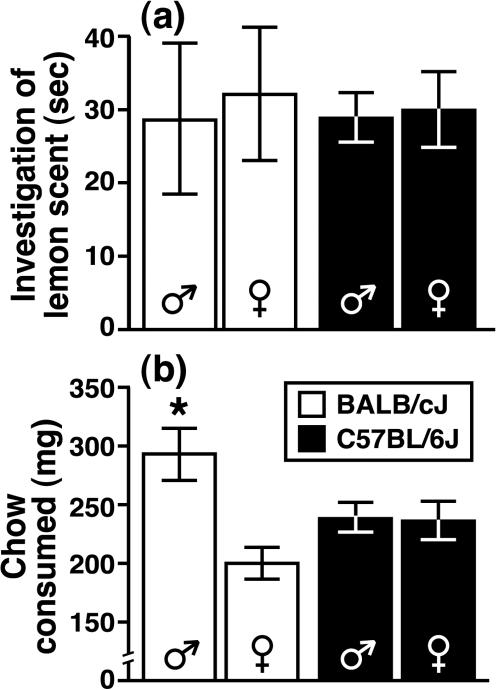
Approach behaviors of adolescent mice towards a novel olfactory stimulus and a food source. After 24 hrs of social isolation, (a) BALB and B6 mice investigated a lemon-scented cotton ball for a similar amount of time. Following complete food deprivation during a 24-hr social isolation period, (b) BALB males consumed more food (standard lab chow) than mice from the other groups during a 10-min period. All data are presented as the mean±SEM (* P<0.05 compared to BALB females and B6 mice of both sexes).

We also assessed the possibility that strain-dependent variation in SI was due to a general difference in the capacity of adolescent mice to express appetitive behavior. Male and female mice (PD 30–36) from the BALB and B6 strains exhibited an equivalent amount of olfactory investigation towards a novel, lemon-scented cotton ball following 24 hrs of social isolation (see *Experiment 5*; **Fig. 4a**; F [1,52] = 0.02, P = 0.90). Two days later, following 24 hrs of food deprivation and social isolation, the mice used during Experiment 5 were tested for consumption of standard laboratory chow (see *Experiment 6*). All mice began to consume the food source within 45 sec and BALB males (PD 32–38) consumed more chow during the 10-min period than mice from all of the other groups (**Fig. 4b**; genotype x gender interaction, F [3,52] = 6.3, P = 0.02).

### Ultrasonic vocalizations during social interactions between early-adolescent mice:

**Figure 5:**
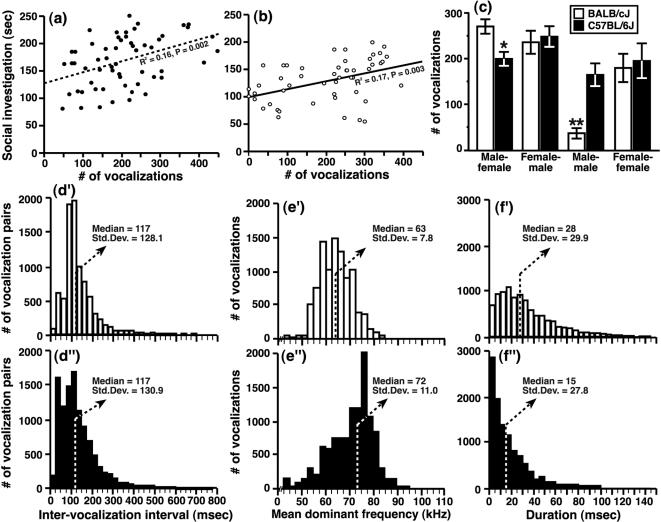
Production and sonographic characteristics of ultrasonic vocalizations during the social interactions of early-adolescent mice. USV emission was positively associated with the SI responses of early-adolescent (a) B6 and (b) BALB mice. (c) USV production was selectively modulated during social interactions that involved a male test mouse from the BALB strain. (d–d′) Emission rates were similar for mice from both strains when USVs were detected. However, compared to BALB mice, the USVs of B6 mice occurred at (e–e′) higher average frequencies and lasted for (f–f′) shorter durations. Data in Figures d–f and d′–f′ are presented as frequency distributions of the raw acoustic signal that was collected during SI tests on PD 30/31. A portion of the data (<0.5% of the sample from each strain) is not illustrated to keep the abscissa of each distribution within a reasonable size for presentation. Data in Figure 5c are presented as the mean±SEM (* P<0.05, ** P<0.01 for BALB vs. B6 mice).

On PD 30/31 (Experiment 1), we recorded ultrasonic vocalizations (USVs) that were produced by early-adolescent mice during SI testing (see Materials and methods). One feature of USV emission, which was common for mice from both strains, entailed a positive association between the total number of vocalizations produced (i.e., ultrasonic ‘syllable’ production, as defined in ref. [53]) and the magnitude of the test mouse SI response (**Figs. 5a & 5b**). Furthermore, for both strains, USV production was more frequent during mixed-sex (mean±standard error of the mean [SEM]; 237±10 USVs) versus same-sex social interactions (149±17 USVs; orthogonal contrast for M-F & F-M vs. M-M & F-F pairs, F [1,103] = 29.7, P<0.0001). Although we did not find an overall difference in USV emission between BALB and B6 mice (main effect of genotype, F [1,103] = 1.5, P = 0.23), there was a strain-dependent sensitivity in USV production that was specific to the sex of mice within an interacting pair (**Fig. 5c**; strain x sex interaction, F [7,103] = 5.4, P = 0.002). For example, compared to M-F pairs from the B6 strain, there were more USVs produced when a female stimulus mouse was introduced to the cage of a BALB male (orthogonal contrast for BALB vs. B6 M-F pairs, F [1,103] = 5.7, P = 0.02). By contrast, USV emission was less frequent for M-M pairs from the BALB strain relative to similar pairs from the B6 strain (see **Figure 5c**; orthogonal contrast for BALB vs. B6 M-M pairs, F [1,103] = 10.4, P = 0.002).

A detailed examination of the sonograms generated for each SI test revealed several, additional strain-dependent differences in the USVs of interacting mice. For example, while the timing of individual syllables within each vocalization-sequence was similar for mice from both strains (**Figs. 5d & 5d′**; χ^2^ = 0.005, d.f. = 1, P = 0.95), the average pitch and duration of individual USVs segregated with the genetic background of mice. USVs produced by B6 mice had a higher frequency (**Figs. 5e & 5e′**; χ^2^ = 2674.4, d.f. = 1, P<0.0001) and shorter duration (**Figs. 5f & 5f′**; χ^2^ = 1401.8, d.f. = 1, P<0.0001) than those of BALB mice. These two particular features of USV emission were also influenced by the sex composition of mice within a pair and its statistical interaction with their collective genetic background (data and statistics not shown).

**Figure 6:**
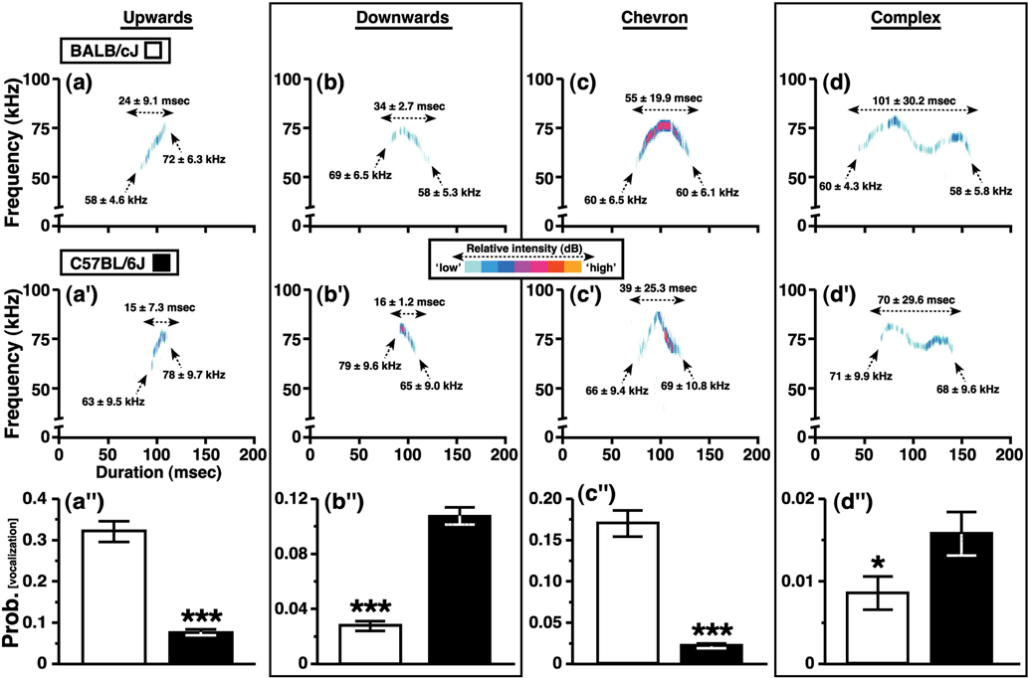
Classification of ultrasonic vocalizations into distinct categories. Representative sonograms and descriptive statistics for different types of USV that were emitted by (a–d) BALB and (a′–d′) B6 mice (see Results for classification criteria). Descriptive statistics (mean±std. dev.) are given for the duration of each call type, as well as the beginning and ending dominant frequency. The USV traces are arbitrarily aligned to the 100-msec demarcation on the abscissa of each sonogram. Intensity changes within each representative vocalization are color-coded. (a″–d″) The production of each USV subtype was strain-dependent (* P<0.05, *** P<0.001 for BALB vs. B6 mice).

**Figure 7:**
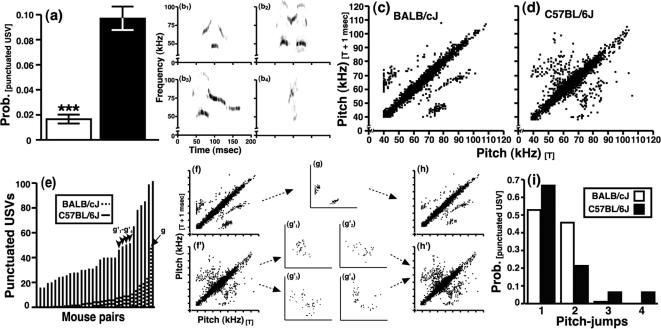
Sonographic characteristics of punctuated ultrasonic vocalizations in relation to the genetic background of mice. (a) B6 mice emitted more punctuated USVs than BALB mice. Four sonographic traces illustrate the varied form of punctuated USVs emitted by early-adolescent mice: (b_1_) a BALB punctuated USV with 2 pitch-jumps, (b_2_) a B6 punctuated USV with 2 pitch-jumps and 2 harmonics corresponding to the 1^st^ and 3^rd^ elements of the vocalization, (b_3_) a B6 punctuated USV with 1 pitch-jump and 1 harmonic corresponding to the 1^st^ element of the vocalization, and (b_4_) a B6 punctuated USV with 1 pitch-jump. (c) 141 BALB and (d) 140 B6 punctuated USVs were graphed as scatter-plots to illustrate the degree of internal frequency modulation present within individual ultrasonic syllables. Data points lying off the diagonal of each plot represent pitch-jumps. (e) Line graph depicting the number of punctuated USVs emitted during a 5-min interaction between mice that expressed the most punctuated USVs of each strain. The arrow and arrowheads denote pairs of mice that emitted a similar number of punctuated USVs. (f–f′) Data re-plotted from Figures 7c and 7d, respectively. (g) The pitch-jumps of punctuated USVs from one extreme BALB pair. (g_1_′–g_4_′) The pitch-jumps of four B6 pairs that produced punctuated USVs at a rate comparable to the BALB pair illustrated in Figure 7g. (h–h′) Data from Figures 7f and 7f′ with pitch-jumps from Figure 7g′ subtracted and the pitch-jumps from Figures 7g_1_′–g_4_′ added, respectively. (i) The total number of pitch-jumps within individual punctuated USVs for each strain graphed as a relative-density histogram.

Although it was not possible to categorize all of the USVs that were present in the sonograms, we did classify 55±20.1% (BALB) and 32±14.5% (B6) of these vocalizations (reported as mean±standard deviation), based on internal pitch-changes, into 5 distinct categories with high reliability between observers (see Materials and methods). USVs were not classified when identification of a clear sonographic pattern was difficult (e.g., extremely short calls were <3 msec) or when the degree of frequency modulation within a call did not fit the criteria outlined below (e.g., some ‘unmodulated’ calls contained single frequency sweeps that were <12.5 kHz). Our scheme for the classification of USVs was as follows: *Upwards*-modulated calls exhibited a continuous increase in pitch that was ≥12.5 kHz, with a final dominant frequency at least 6.25 kHz greater than the initial pitch (see **Figures 6a & 6a′**). *Downwards*-modulated calls exhibited a continuous decrease in pitch that was ≥12.5 kHz, with a terminal dominant frequency at least 6.25 kHz less than the pitch at the beginning of the vocalization (**Figs. 6b & 6b′**). The general sonographic profile of *chevron*-classified calls resembled an ‘inverted-U’, which was identified by a continuous increase in pitch ≥12.5 kHz followed by a decrease that was ≥6.25 kHz (**Figs. 6c & 6c′**). USVs were classified as *complex* if one syllable contained two or more directional changes in pitch, each ≥6.25 kHz (**Figs. 6d & 6d′**). A fifth USV category, *punctuated* calls, was characterized by discontinuous jumps in frequency that were ≥7.5 kHz. We identified these pitch-jumps as ‘punctuations’ because they occurred rapidly (≤1 msec), dividing each ultrasonic syllable into two or more distinct elements (see **Figures 7b_1_–7b_4_**).

We detected strain-dependent effects on the probability of producing calls from each of the five classes of USV. For example, early-adolescent BALB mice emitted more upwards-modulated USVs than B6 mice (**Fig. 6a″**; main effect of genotype, F [1,103] = 125.5, P<0.0001), whereas age-matched B6 mice expressed downwards-modulated USVs more frequently than BALB mice (**Fig. 6b″**; main effect of genotype, F [1,103] = 109.8, P<0.0001). Chevron-call production was relatively common during the social interactions of BALB mice, but not B6 mice (**Fig. 6c″**; main effect of genotype, F [1,103] = 142.4, P<0.0001). Furthermore, although the production of complex calls was less common than vocalizations from other USV subtypes, B6 mice emitted more of these calls than BALB mice (**Fig. 6d″**; main effect of genotype, F [1,103] = 6.1, P = 0.02).

The production of upwards-, downwards- and chevron-classified USVs also varied as a function of the sex of mice within a pair and its interaction with their genetic background (P<0.05 for the sex and genotype x sex interaction factors, data and F-statistics not shown). Interestingly, genetic background was the only variable that influenced complex call production during social interactions between early-adolescent mice (see **Figure 6d″**; P>0.05 for the sex and genotype×sex interaction factors, data and F-statistics not shown). Similarly, punctuated calls were influenced by the genetic background of mice (**Fig. 7a**; main effect of genotype, F [1,103] = 48.6, P<0.0001), but not by the sex of mice or its interaction with genetic background (P>0.05 for both factors, as reported above).

A strain difference in the sonographic pattern of punctuated USVs was evident when the dominant frequencies of selected calls were plotted at *t* (the x-axis in **Figures 7c & 7d**) and contrasted to changes in pitch within the same call that occurred at *t*+1 msec (the y-axis in **Figures 7c & 7d**). As illustrated in **Figures 7c and 7d**, data points near the diagonals have a paired-pitch ratio (pitch_[t]_/pitch_[t+1]_) approximating 1.0 and thus represent continuous changes in the dominant frequency (or pitch) of a call. Paired-pitch ratios deviating from 1.0 aligned off the diagonals and represent discontinuous breaks in the dominant frequency of a call (these punctuations were tallied as ‘upwards’ [pitch_[t+1]_≥(pitch_[t]_+7.5 kHz)] or ‘downwards’ [pitch_[t+1]_≤(pitch_[t]_+7.5 kHz)] oriented pitch-jumps, as outlined above). The punctuated calls of BALB mice were dominated by three distinct, paired-pitch ratios (note the three data ‘clouds’ lying off the diagonal in **Figure 7c**). By contrast, a much less stereotyped pattern of pitch-jumps was evident for the punctuated calls of B6 mice (see **Figure 7d**). Since the emission of punctuated USVs was less common during social interactions involving BALB mice (see **Figure 7a**), greater sampling of the BALB sonograms was required to attain a similar number of paired-pitch ratios for each strain (≈59% of BALB [n = 141] and ≈10% of B6 [n = 140] punctuated USVs are plotted in **Figures 7c & 7d**, respectively). Therefore, to examine whether this apparent strain-dependent pattern had resulted from a sampling bias, we sampled additional punctuated USVs from select pairs of mice. Pitch-jumps from random samples (**Figs. 7f & 7f′** re-illustrate data that are presented in **Figures 7c & 7d**, respectively) were contrasted to pitch-jumps from SI tests that we matched for the production of punctuated USVs *a posteriori* (arrow *g* and arrowheads *g_1_′–g_4_′* in **Figure 7e** denote pairs of mice that emitted a comparable number of punctuated calls). The strain-dependent pattern in paired-pitch ratios was still readily apparent under conditions in which the pitch-jumps of one particularly extreme BALB pair (**Fig. 7g**) were removed from the plot (**Fig. 7h**) and when all of the pitch-jumps produced by 4 comparable B6 pairs (**Figs. 7g_1_′–7g_4_′**) were included (**Fig. 7h′**). Additionally, although a majority of the punctuated USVs emitted by early-adolescent mice contained 1–2 pitch-jumps, which corresponded to 2–3 distinct elements per syllable, approximately 15% of the B6 punctuated calls included 3–4 pitch-jumps, as illustrated in **Figure 7i**.

A set of tests for correlation revealed several additional relationships between USV emission and the SI responses of early-adolescent test mice (**Table 2**). While the total number of USVs produced was positively associated with SI responses of test mice from both strains (see **Figures 5a & 5b**), other patterns among USV characteristics and SI were strain-specific. Interestingly, longer calls predicted larger SI responses by BALB test mice (but not B6 mice), whereas higher-pitched calls predicted greater social responsiveness for B6 test mice (but not BALB mice).

**Table 2:** Pearson's correlation coefficients relating SI responses of test mice to variables associated with USV emission (* P<0.05, ** P<0.01, *** P<0.001).

USV variable	BALB/cJ SI	C57BL/6J SI
N _[USV]_	0.43**	0.40**
Pitch (kHz)	0.15	0.53***
Duration (msec)	0.58***	0.10
N _[upwards USV]_	0.07	0.04
N _[downwards USV]_	0.58***	0.52***
N _[chevron USV]_	0.53***	0.08
N _[complex USV]_	0.45**	0.03
N _[punctuated USV]_	0.30*	0.22

### Strain-dependent variation in the rewarding aspects of social interaction:

**Figure 8:**
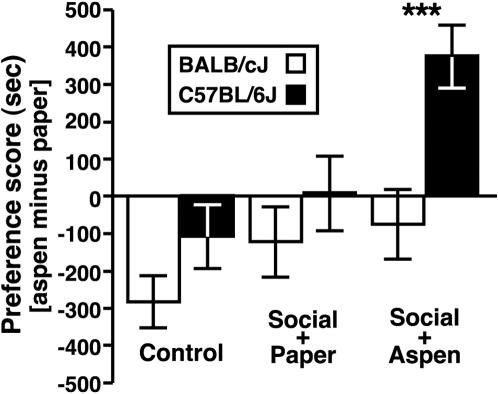
Strain-dependent variation in the social conditioned place preference responses of early-adolescent mice. Unconditioned mice from both strains expressed a preference for the paper bedding (control bars) and this natural bias obscured any conditioning effect that might have resulted from pairing social interaction with paper bedding (social *plus* paper bars). However, there was a robust, strain-dependent SCPP response for B6 mice when the less-preferred aspen bedding was paired with social enrichment (social *plus* aspen bars). Preference scores were calculated as the time each mouse spent in the aspen bedding-lined environment *minus* the duration in the paper bedding-lined compartment of the place-preference arena. All data are presented as the mean±SEM (*** P<0.001 for BALB vs. B6 mice).

To assess whether the strain-dependent difference in SI between early-adolescent mice was related to the rewarding nature of social interaction, we conducted an experiment using a social conditioned place preference (SCPP) procedure (see *Experiment 7*). Consistent with SCPP responses of early-adolescent B6 mice that were exposed to a protocol which entailed 24-hr home cage conditioning sessions (see ref. [51]), in the present study, PD 30–35 mice from the B6 strain expressed a robust place-preference response for an aspen bedding-lined environment after it had been associated with social interaction during three daily 30-min conditioning sessions (**Fig. 8**; main effect of conditioning group, F [5,82] = 7.5, P<0.001). By contrast, age- and experience-matched BALB mice were much less responsive to the same conditioning contingency (between-strain orthogonal contrast, F [1,82] = 3.5, P<0.001). The sex of mice (F [1,82] = 2.1, P = 0.15), nor its interaction with the particular conditioning-group (F [5,82] = 0.3, P = 0.89), had an influence on the place preference responses of adolescent mice.

Of note, repeated exposure of early-adolescent mice to each environment (aspen and paper bedding), *in the absence of social conditioning*, revealed a natural preference for paper bedding (*t* = 4.8, d.f. = 39, P<0.0001 compared to H**_Ø_** = 0) for mice from both strains (between-strain orthogonal contrast, F [1,82] = 1.6, P = 0.12). Within the context of social conditioning, therefore, a strain-dependent difference in SCPP was undetectable when paper bedding had been associated with social enrichment (between-strain orthogonal contrast, F [1,82] = 0.9, P = 0.37). However, as mentioned above, early-adolescent B6 mice—unlike age-matched BALB mice—spent substantially more time within an otherwise negative environment (aspen bedding) once it had been associated with opportunity for social interaction (see **Figure 8**).

## Discussion

### Social approach

Adult female mice can give birth to a new litter at approximately three-week intervals under favorable conditions. With the emergence of new offspring, maternal behaviors towards the older litter declines and juveniles are faced with the challenge of living more independently as adolescents. This period of *early adolescence* is a developmental stage when juveniles shift their social attention away from maternal care and towards peers [19]. In feral populations, young animals, including mice, experience a particularly high level of mortality after weaning [29], [39], [54], [55], possibly due to the inherent risks of traveling further from the nest. Trapping studies have also demonstrated that adolescent mice are captured in pairs more frequently than adults [56], which suggests that young mice may travel in social groups while dispersing. A heightened level of gregariousness among peers is in fact one of the most consistent findings regarding studies of adolescent behavior in mammals [8], [20], [22], [24], [32], [33], [57]. That robust forms of social interaction are a regular occurrence among prepubescent animals implies that some factor other than reproductive interests is the primary motivation to engage in such aspects of sociality. While adolescence includes the pubertal stages of development, it also encompasses the more gradual acquisition of psychosocial skills that precede, accompany and follow the capacity to produce offspring [18], [34], [36]. Moreover, later stages of adolescence are distinguishable from early adolescence and adulthood by differences in social behavior [20], [22], [24], [58], responsiveness to environmental or pharmacological manipulations [37], [59]–[62] and hormone sensitivity [41], [63], [64].

Consistent with this view of adolescent development, we found that the motivation of early-adolescent B6 mice to investigate a conspecific was unresponsive to the particular sex of the individual they were paired with (i.e., the SI responses of B6 test mice from all test-by-stimulus combinations were similar on PD 25/26). As mice matured, however, their social interests became more sensitive to the sexual identity of the respective stimulus mouse. Male test mice from the B6 strain, for example, expressed larger SI responses towards females on PD 45/46, while age-matched B6 females investigated other females more than males. By contrast, sex-selective patterns of social approach were evident for BALB mice on PD 25/26, as male test mice displayed a higher level of social interest in females relative to males. Moreover, as BALB males matured, an additional increase in the bias towards investigating females was evident. Overall, our results thus demonstrate a strain-dependent influence on the social approach behaviors of early-adolescent mice, which becomes increasingly obscured by the emergence of sex-selective social interests in older mice.

Previously reported differences in the social tendencies of adult BALB and B6 mice include a low level of parental care by maternal BALB females, and both decreased sexual motivation and increased aggressive tendencies among adult BALB males (see ref. [49] for a review). Our preliminary studies indicated that, when given a sufficient amount of time to interact (i.e., 10 min instead of the 5-min duration that was used for Experiment 1), tail rattling and wrestling behaviors occurred in approximately 35% of M-M interactions that involved BALB mice (PD 39–42), but never during the social interactions of age-matched pairs of B6 males. Furthermore, five days before it became evident for similarly paired BALB males, mounting behaviors occurred during approximately 25% of the social interactions in which B6 test males had been paired with a female stimulus mouse. These patterns of social behavior lacked the overall intensity that is typical of sexual and aggressive behaviors between adult mice. For example, injuries were never observed on males that had been tested and housed together throughout adolescence, while mounting attempts were directed at the sides and front of a female as often as they were targeted to the flank, and lordosis never occurred. These findings are consistent with a view of adolescent development in which a relatively strong and generalized form of social motivation is gradually supplanted by behaviors that may take on a function once puberty occurs. Importantly, we found that the strain-dependent difference in SI was in fact smaller (F-F pairings) or undetectable (M-F, F-M & M-M pairings) for mice that were tested on PD 45/46. Together with our control experiments (see Experiments 2–6), the data strongly suggest that strain-dependent variation in the social approach phenotypes of early-adolescent mice is attributable to a genetic influence on a specific, social-motivational process.

### Ultrasonic Vocalizations

Measurements of USV production among early-adolescent mice complemented the strain difference that was found for SI. For example, when the test mouse was a BALB male, the number of USVs emitted during social interaction was particularly sensitive to the sexual identity of the stimulus mouse. Despite this strain-dependent difference, the quantity of USVs during social interaction was a strong, positive predictor of the extent that test mice from both strains would investigate the stimulus mouse. This finding is consistent with studies in rats, which suggest that USVs reflect social affect [65]. The rate of USV emission by early-adolescent mice in the present study (2.7 calls/sec collapsed across strain, gender and time-into-trial) was very similar to what has been previously reported for mice during contexts such as mating [66], [67] or infant social-isolations [68]. Interestingly, infant mice from the B6 strain [69], [70] and the related C57BL/10J strain [68] tend to emit *fewer* USVs than BALB mice upon maternal separation, although infant B6 mice still appear to produce USVs of a shorter duration and higher frequency [68]. To our knowledge, the present study provides the first demonstration of USV emission by mice outside of social contexts that are related to aggression, mating or mother-infant attachments.

Aside from the twenty-two- and 50-kHz USVs produced by young rats [71] and the lower-frequency distress calls of mouse pups [72] few studies have evaluated the sonographic features of rodent vocalizations within a behavioral context (also see refs. [53], [66], [68], [73]). Consistent with a recent study of song production by adult mice [53], we found many USVs that were frequency-modulated, occurring in repetitive bouts separated by periods of silence. These vocalizations were remarkably complex, with a significant effect of genotype on each distinct USV category that was classified. Interestingly, the genetic background of mice within an interacting pair was the sole influence on the production of complex and punctuated USVs, which is topically similar to our behavioral findings for early-adolescent B6 mice. Furthermore, the pitch-jumps which characterized the sonographic profile of punctuated USVs were much more variable for vocalizing B6 mice relative to BALB mice. It is thus intriguing to consider whether specific categories of vocalization may be functionally related to the strain-dependent difference in SI among early-adolescent mice; however, an assessment of social approach in the context of USV play-backs will be necessary to fully evaluate this possibility.

### Social Reward

We also found a strain difference in the reward value that early-adolescent mice assigned to the opportunity for social interaction. That young B6 mice found social contact rewarding is consistent with our previous work [51]; however, it extends those findings by demonstrating that a much shorter period of social interaction (i.e., 30 min during the present study vs. 24 hrs during our previous study) can also serve as an effective conditioning stimulus. Our results thus indicate that some aspect of social contact, which occurs within the first 30 min of reunion with conspecifics, can serve as a powerful reward for early-adolescent B6 mice. By contrast, age-matched BALB mice in the present study did not express a SCPP response after a series of three daily 30-min conditioning sessions nor do they following ten contiguous conditioning sessions that alternate between 24-hr periods of social enrichment and social isolation [51]. It is important to mention one notable difference between the present findings and our previous SCPP study. Unlike our previous experiments, we found that unconditioned mice from both the BALB and B6 strains expressed a natural preference for the paper bedding. Although this difference will require additional attention in future experiments, one possible contributory factor is the amount of experience that mice had with the aspen and paper beddings (i.e., mice had spent a total of 1.5 hrs with each bedding prior to testing vs. 120 hrs with each bedding in our previous study).

Classical theories of motivation [74]–[76] propose that approach behaviors are indicative of a state of reward and that neutral stimuli can gain value through association with these rewarding experiences [77], thereby extending the range of reward-related opportunities that are available to an individual. In the present study, we have shown that early-adolescent B6 mice are particularly motivated to approach conspecifics, and while doing so, produce USVs at a high rate. The B6 social phenotype is most distinct from that of BALB mice during early adolescence, as the strain difference gradually diminishes with the concurrent emergence of sex-specific social approach by mice that are approaching puberty. These findings are consistent with previous studies that have underscored the fundamental importance of ‘timing’ for identifying the physiological [37], [78] and gene-regulatory [79], [80] processes that underlie changes in behavior (also see refs. [81], [82]).

Well-defined genetic influences, which are operational during a specific stage of behavioral development, provide a straightforward mechanism through which phenotypic variation can respond to selection pressures that arise predictably during an animal's lifetime. The nature of social approach among early-adolescent B6 mice is consistent with observations that demonstrate social interactions between young mammals can be amicable [8], [22], [33], involving unrelated individuals [83]–[85], and possibly functioning to mitigate the risks associated with dispersal [86]. We propose that the affiliative social interactions of young mice are driven by a state of reward and that differences in the nature of these interactions can be moderated by genetic factors during a relatively narrow window of adolescent development.
